# Corneal Tonometric and Morphological Changes in Patients with Acromegaly

**DOI:** 10.3390/jcm11226750

**Published:** 2022-11-15

**Authors:** Izabela Skrzypiec, Joanna Wierzbowska, Maria Sobol, Grzegorz Zieliński

**Affiliations:** 1 Department of Ophthalmology, Military Institute of Medicine—National Research Institute, 128 Szaserow Str, 04-141 Warsaw, Poland; 2 Department of Biophysics, Physiology and Pathophysiology, Medical University of Warsaw, 5 Chalubinskiego Str, 02-004 Warsaw, Poland; 3 Department of Neurosurgery, Military Institute of Medicine—National Research Institute, 128 Szaserow Str, 04-141 Warsaw, Poland

**Keywords:** acromegaly, cornea, intraocular pressure, tonometry, anterior eye segment, corneal endothelium, growth hormone

## Abstract

**Highlights:**

- This study is the first prospective clinical trial to evaluate the anterior corneal parameters in patients with acromegaly before and after transsphenoidal pituitary adenoma resection on the basis of several months of postoperative follow-up.
- An increase in CD and a decrease in AVG and CCT_NSM_ were observed after pituitary adenoma resection.
- The mean CCT measured both with an ultrasonic pachymeter and with non-contact specular microscopy was significantly lower as compared to the preoperative values.
- IOP decreased statistically for the measured IOP air puff values. A downward trend was observed after treatment in the case of IOP GAT and IOPc, but statistical significance was not achieved.
- Statistically significant reduction in CH and CRF was found after the surgical treatment of acromegaly.

**Abstract:**

(1) Purpose: This study aimed to investigate the changes in Reichert Ocular Response Analyzer (ORA) parameters, corneal endothelium parameters, central corneal thickness (CCT), and intraocular pressure (IOP) before and after the transsphenoidal resection of pituitary adenoma in patients with acromegaly. (2) Methods: This was a single-center, prospective, interventional study. Twenty patients with newly diagnosed acromegaly were examined before and 19 ± 9 months after transsphenoidal resection. The participants underwent a comprehensive ophthalmological examination including pneumatic IOP (IOP air puff), Goldmann applanation tonometry (IOP GAT), CCT measured using the iPac pachymeter (CCT_UP_), IOP value corrected for CCT_UP_ using the Ehlers formula (IOPc) ORA measurements included corneal hysteresis (CH), corneal resistance factor (CRF), corneal-compensated IOP (IOPcc), and Goldmann-correlated IOP (IOPg). CCT from non-contact specular microscopy (CCT_NSM_), the number of endothelial cells (CD) per mm2, and average cell size (AVG) were determined with non-contact specular microscopy. (3) Results: A statistically significant decrease was observed in CCT_UP_ (*p* = 0.007), and IOP air puff (*p* = 0.012) after surgery. Moreover, we noted a statistically significant increase in CD (*p* = 0.001), and a statistically significant decrease in AVG (*p* = 0.009) and CCT_NSM_ (*p* = 0.004) after surgery. A statistically significant decrease was also observed in IOPg (*p* = 0.011), CH (*p* = 0.016), and CRF (*p* = 0.001) after surgery. The mean value of IOP GAT and IOPc was lower after the surgery. However, the difference was not statistically significant. (4) Conclusions: Our study revealed significant changes in biomechanics, corneal endothelium, CCT and IOP after pituitary adenoma resection in patients with acromegaly. It proves that the eye might be sensitive to long-term overexposure to growth hormone (GH) and insulin-like growth factor-1 (IGF-1). We suggest that disease activity be taken into consideration on ophthalmological examination.

## 1. Introduction

Acromegaly is a rare chronic disease caused by the excessive secretion of growth hormone (GH), most often by pituitary somatotroph adenoma [1]. The excessive secretion of GH stimulates the liver to produce growth factors responsible for the symptoms of the disease, including insulin-like growth factor-1 (IGF-1). It leads to the hypertrophy of soft tissues, changes in appearance, and the development of organ complications contributing to the shortening of the duration and lowering the quality of life [1,2]. The first-line treatment for acromegaly includes the transsphenoidal resection of the pituitary adenoma (after an initial treatment with somatostatin analogues), which is associated with a 50–70% chance of complete recovery and depends on the surgeon’s experience, invasiveness of the procedure and tumor size [3]. Ophthalmic changes in acromegaly depend on the compression and infiltration of the pituitary somatotroph adenoma on the optic chiasm and surrounding structures (mass effect) and on the symptoms associated with the effect of GH-stimulated excess IGF-1 on the eye tissue [4,5,6]. The effects of GH and IGF-1 on the ocular tissues in acromegalic patients have not been identified in detail yet. IGF receptors are present in all the layers of the cornea, and IGF plays an important role in regulating the homeostasis of the corneal tissue, e.g., wound healing [7]. Animal studies showed the stimulatory effect of IGF-1 on the production of scleral extracellular matrix, which resulted in increased resistance and disturbed outflow of aqueous humor [8].

Ocular findings, which might be the due to GH and IGF-1 activity, occurring in the anterior segment of the eye in acromegalic patients may be associated with an increased central corneal thickness (CCT) [9,10,11,12,13,14], increased intraocular pressure (IOP) [9,10,11,13,15], changes in corneal biomechanics [10,16], a decrease in endothelial cell density [17], a narrower angle of the anterior chamber [11], and dry eye syndrome related to meibomian gland dysfunction [11,18]. All previous studies concerning ocular changes in the anterior segment of the eye presented the comparison between patients with acromegaly and healthy control group patients. However, available results were inconsistent in terms of the presence of differences in CCT values [15,16,19,20], IOP [12,14,19,20,21] and corneal biomechanical parameters [20] between those two groups of subjects.

The aim of our study was to evaluate changes in the anterior segment of the eye—including the biomechanics, endothelial assessment, CCT and IOP before and after transsphenoidal pituitary adenoma resection. To date, no similar prospective analysis of tonometric, morphological and biomechanical parameters has been performed before and after treatment in a group of patients with this rare disease.

## 2. Materials and Methods

It was a prospective, interventional study, conducted at the Military Institute of Medicine in Warsaw, Department of Ophthalmology with the contribution of the Department of Neurosurgery. The study group consisted of twenty adults of both sexes, newly diagnosed with acromegaly. The diagnosis of acromegaly was made according to the Endocrine Society Guidelines criteria and confirmed with the examination of surgically resected tissues [22]. All patients received initial treatment with somatostatin analogues 3–6 months prior to the surgery. The patients who did not achieve remission (defined as normal GH and IGF-1 values after surgery) continued maintenance therapy with somatostatin analogs from 20 to 40 mg/mL (octreotide long-acting release; Sandostatin LAR, Novartis Pharmaceuticals, Basel, Switzerland). The ophthalmological examinations were performed one day before and 19 ± 9 months after the transsphenoidal resection of the pituitary adenoma performed by the same experienced neurosurgeon [G.Z.]. All individuals with significant refractive errors (spherical equivalent (SE) > +/− 4.0 Dsph), best corrected visual acuity (BCVA) < 0.8, glaucoma, retinal, sclera or corneal diseases, patients who had previous ocular surgery or trauma, with a history of contact lens use, chronic topical or systemic steroid therapy, current or former smokers and alcohol abusers were excluded of the study. All subjects were informed about the study procedure, and written consent was obtained. The study followed the tenets of the Helsinki Declaration and was approved by an institutional ethics committee (approval No. 76/WIM/2016).

### Examination Protocol and Measurements

Serum GH, IGF-1, glucose levels, height and weight to calculate Body Mass Index (BMI), disease duration and dimensions (height) of pituitary adenoma on MRI examination. Disease duration was defined as the time from the onset of acromegaly symptoms to the clinical diagnosis. All patients underwent histopathological confirmation of GH-secreting pituitary adenoma.

All enrolled patients underwent a detailed ophthalmological examination performed by the same ophthalmologist (I.S.) before and after the surgery at the same time of the day. Autorefractometry and pneumatic intraocular pressure (IOP air puff) (Auto kerato-refracto-tonometer TRK-2P; Topcon, Tokyo, Japan), SE (calculated as Cylinder/2 + Sphere), Snellen BCVA, Goldmann applanation tonometry (IOP GAT), anterior segment and fundus examinations, ultrasonic pachymetry (CCT_UP_), non-contact specular microscopy, and ocular response analyzer measurements (ORA) were recorded for all the participants. To avoid recording daily IOP, ORA and CCT fluctuations, the measurements were performed at the same time of the day, between 8 am and 10 am as a part of the study consultation. All contact procedures were performed at the end of the examination, keeping time intervals to exclude their influence on the obtained results. The IOP GAT value was corrected for CCT_UP_ using the Ehlers formula (IOPc) [23]. CCT_UP_ was measured with an ultrasonic pachymeter (UP) (iPac^®^; Reichert Ophthalmic Instruments Inc., New York, NY, USA) that had been calibrated prior to taking the measurements. Local anesthesia with 0.5% proxymetacaine hydrochloride (Alcaine; Alcon, Puurs, Belgium) was used. CCT_UP_ was measured in patients sitting upright and looking straight at a distant target. The UP probe was sterilized and positioned perpendicularly and centered on the pupil as precisely as possible. Three consecutive measurements were taken, from which the average was calculated. Non-contact specular microscopy (EM4000; Tomey Corporation Inc., Nagoya, Japan) was used to evaluate the CCT (CCT_NSM_) and corneal endothelium morphology. The patient was instructed to look straight ahead at the fixation target. The best quality image was automatically selected and the analysis of cell parameters was performed by the built-in software, with the results being finally displayed on the screen. The corneal endothelial parameters included the number of endothelial cells (CD) per mm^2^, and average corneal endothelium cell size (AVG). ORA Reichert (ORA; Reichert Ophthalmic Instruments, New York, NY, USA) measurements included corneal hysteresis (CH), corneal resistance factor (CRF), corneal-compensated intraocular pressure (IOPcc), and Goldmann-correlated intraocular pressure (IOPg). Four high-quality measurements were made, and the average was calculated. There was a 15 s break between the measurements.

Statistical analysis was performed using the Statistica 13.1 package. To carry out statistical analysis, the right or left eye was randomly chosen with the coin-toss method for each patient. For a group of 20 patients, we received at least 73% power to detect differences of at least 10% at the level of α = 0.05. The quantitative variables were summarized using descriptive statistics: the mean, standard deviation, median and range, and upper and lower quartile. The distribution of each variable was tested for consistency with the normal distribution using the Kolmogorov–Smirnov test. The categorical (sex, remission) data were presented as percentages. The nonparametric Wilcoxon pair test was used to evaluate statistically significant differences before and after surgery. A correlation analysis was performed in order to verify the correlation between the analyzed parameters and disease duration, tumor size (height), IGF-1, GH, glucose, and BMI. Since at least one of the analyzed variables did not meet the condition of the normal distribution, Spearman’s rank coefficient was determined. The results were considered statistically significant with *p* < 0.05. Two multiple regression models were used in order to evaluate relationships between CCT values as a dependent variable and IGF-1, glucose, and the duration of acromegaly. In the first model glucose and IGF-1, while in the second one the duration of the disease and IGF-1 were considered independent variables.

## 3. Results

### 3.1. Demographic Characteristics

Twenty eye examinations of acromegaly patients were prospectively assessed. The mean age of the group was 49.7 ± 13.4 years with a median of 49 years (range of 29–75 years). There were 13 (65%) women and 7 (35%) men in the group of patients. The duration of acromegaly was 8.3 ± 5.2 years, the median was 7 years (range: 2–20 years). The pituitary tumor was completely removed in 14 patients. Fifteen patients achieved remission after surgery and five continued maintenance therapy with somatostatin analogs. Before surgery, four patients had been diagnosed with arterial hypertension and five patients with diabetes type II—the oral treatment remained unchanged before and after surgery. The characteristics of the group including information before and after surgery and the mean values, standard deviation (SD), median and range for all of the variables are presented in Table 1.

A statistically significant decrease was observed in the level of IGF-1, GH and glucose after surgery. There was no statistically significant change in BCVA or SE. A statistically significant decrease was observed in IOP air puff after surgery. The mean value of IOP GAT and IOPc was lower after surgery. However, the difference was not statistically significant (Table 1).

### 3.2. Specular Microscopy

Statistically significant differences were found for all considered variables. A statistically significant increase in CD (*p* = 0.001) occurred from the median of 2346 cells/mm^2^ before surgery to 2532 cells/mm^2^ after surgery. In the case of AVG, a statistically significant decrease was noted, with median values of 416 μm^2^ and 395 μm^2^ before and after surgery, respectively. A statistically significant decrease was also observed in CCT_NSM_. Before surgery, the median CCT_NSM_ was 563 µm, which then was reduced to 542 μm after surgery. No significant correlation was determined between CD, AVG, CCT_NSM_ and disease duration, tumor size, IGF-1, glucose and BMI either before or after surgery. The descriptive statistics and *p*-value for all considered parameters are presented in Table 2.

### 3.3. ORA

Statistically significant differences were observed in the IOPg, CH, and CRF parameters. A statistically significant decrease occurred in all of those parameters after surgery. Before surgery, the medians were 14.9 mmHg (IOPg), 10.8 mmHg (CH) and 10.3 mmHg (CRF), while after surgery the medians were 14.2 mmHg, 10.0 mmHg and 9.7 mmHg, respectively. No statistically significant difference was found for IOPcc (*p* = 0.314) (Table 3). A moderate statistically significant positive correlation occurred between tumor size and CRF and CH, and a moderate positive correlation was observed between IGF-1 and CH prior to surgery. The values of Spearman’s rank coefficient were 0.596, 0.476 and 0.634, respectively. In addition, there was a strong statistically significant positive correlation between CRF and glucose level (ρs = 0.623), and a moderate positive correlation between CRF and tumor size (ρs = 0.573) and CRF and IGF-1 (ρs = 0.548). After surgery, a moderate statistically significant positive correlation was noted between IOPg and glucose level (ρs = 0.515) (Table 4). Correlation analyses between GH, IGF-1, glucose, tumor height and ORA before and after treatment in the study group are presented in Table 4.

### 3.4. Ultrasonic Pachymetry

**Figure 1:**
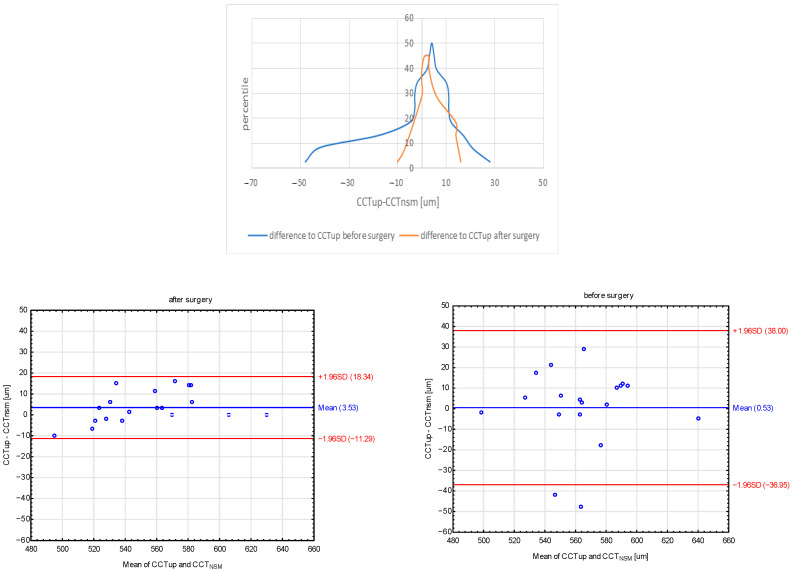
Mountain plots and Bland–Altman plots showed the distribution of the differences between CCT_UP_ and CCT_NSM_ before and after surgery. CCT_UP_, ultrasonic pachymetry; CCT_NSM_, non-contact specular microscopy.

A statistically significant decrease was found in CCT_UP_ (*p* = 0.007). Before surgery, the median CCT_UP_ was 565 μm (range from 498 μm to 638 μm) which was reduced to 562 μm (range from 490 μm to 630 μm) after surgery (Table 1). No statistically significant correlation was noted between CCT_UP_ and disease duration, tumor size, IGF-1, glucose, BMI, and systolic and diastolic pressure either before or after surgery. The mean CCT measurements were higher in UP (CCT_UP_) than in specular microscopy (CCT_NSM_) both during the ophthalmological visit before and after surgical treatment. The mean CCT difference was 0.53 μm (range from 38.8 μm to 36.95 μm) before and 3.53 μm (range from 18.34 μm to −11.29 μm) after surgery (Figure 1).

The independent factors were not found to be statistically significant (*p* > 0.05) in any of the models of multivariate analysis.

CCTmodel_1 = (25.8 ± 0.13.8) × glucose + (0.037 ± 0.029) × IGF-1 + (415 ± 72)

CCTmodel_2 = (26.3 ± 14.0) × glucose + (−1.91 ± 1.77) × duration_of_acromegaly + (437 ± 71)

## 4. Discussion

To our knowledge, this study is the first prospective clinical trial based on several months of postoperative follow-up conducted to evaluate biomechanical, tonometric and morphological corneal parameters in acromegaly patients undergoing transsphenoidal pituitary adenoma resection.

Numerous authors suggested that the eye might be sensitive to IGF-1 and GH levels, especially in the active phase of the disease. We hypothesized that the resection of pituitary gland adenoma releasing excessive GH might affect some parameters of the cornea, which has viscoelastic properties. Available literature lacks information on the impact of pituitary adenoma surgery on corneal parameters.

The study revealed statistically significant differences for the majority of the analyzed parameters at the end of the mean of the 19-month observation. The mean number of endothelial cells (CD) per mm2 significantly increased and the mean average corneal endothelium cell size (AVG) significantly decreased following the surgery. Furthermore, the mean central corneal thicknesses measured both with an ultrasonic pachymeter and with non-contact specular microscopy were significantly lower as compared to the preoperative values. Finally, a statistically significant decrease in corneal biomechanical parameters was observed after the intracranial surgery.

### 4.1. Corneal Endothelial Cell Parameters

Our study demonstrated an increase in CD and a decrease in AVG and CCT_NSM_ after pituitary adenoma resection. A statistically significant increase in CD occurred from the median of 2346 cells/mm^2^ before surgery to 2532 cells/mm^2^ after surgery. It remains unclear why we observed a CD increase after surgery in our study. The above changes are probably related to the influence of growth factors on the corneal endothelial cells. On the one hand, GH mediates cell proliferation and growth via IGF-1, and on the other hand, it accelerates the aging process and apoptosis, which is specific to various tissues and cells. The protective role of IGF-1 against apoptosis through mitochondria, which are abundant in the corneal endothelial cells, was also defined [24]. Patients with acromegaly have an increased amount of extracellular fluid, which is related to the sodium-retaining effect of GH/IGF-1 [23]. Achieving biochemical disease control in acromegalic patients may result in water reduction in the stromal structures (CCT reduction) and an improvement in the endothelial osmotic pump (ATPase Na+/K+), maintaining the state of relative stromal dehydration. Nevertheless, further studies on a larger group of acromegalic patients and longer observations are necessary to confirm this result. The lack of correlation between GH/IGF-1 and endothelial parameters may indicate that it may act in synergy with other growth factors or in an autocrine or paracrine manner.

Thus far, only one study published by Hatipoglu et al. analyzed changes in endothelial cell density and morphology in patients with acromegaly [17]. The observations included no differences in CCT, lower endothelial cell density and greater endothelial cellular area in the acromegalic group compared to healthy subjects, which correlated with the duration of the disease [17]. In addition, the researchers divided the study group into two subgroups—the active and inactive forms of the disease. They did not observe any differences in the density and morphology of endothelial cells either [17]. In the study, the duration of acromegaly was considered a risk factor for corneal endothelial cell damage [17]. Due to the small size of the group with the active form of the disease, it is impossible to make a similar comparison in our study, which is one of the limitations of our study.

### 4.2. Biomechanical Properties of the Cornea

ORA is a non-invasive method of assessing the biomechanical properties of the cornea. This device measures IOPg and IOPcc. It also analyzes CH, i.e., the ability to absorb and dissipate energy, and CRF, i.e., determining the total resistance of the cornea to the applied force. The use of ORA was associated with greater repeatability, suggesting a more reliable IOP assessment [25].

Our study demonstrated a statistically significant reduction in CH and CRF after the surgical treatment of acromegaly, which means that changes in corneal biomechanics might be reversible upon treatment. The biomechanical properties of the cornea may change in systemic diseases and diseases associated with hormonal fluctuations [26,27]. To date, three separate studies have assessed the biomechanical properties of the cornea in acromegalic patients compared to healthy controls [10,16,20]. Altinkaynak et al. and Ozkok et al. reported higher values of CH and CRF in acromegalic patients compared to healthy controls [10,16]. Contrary to those studies, Sen et al. observed no differences in CH, CRF, IOPcc and IOPg between the acromegaly group compared to healthy subjects, which, according to the researchers, might be due to the low percentage of uncontrolled acromegaly subjects in their sample. IGF-1 has a well-known effect on proteoglycans, corneal extracellular matrix synthesis, and keratocyte metabolism [28]. Increased IGF-1 values in acromegaly probably affect the viscoelastic properties of the cornea by increasing CH and CRF.

Our study revealed a significant reduction in IOPg (from the median of 14.9 mmHg before surgery to 14.2 mmHg after surgery) in contrast to IOPcc, where no significant differences were determined. Altinkaynak et al. and Ozkok et al. found higher IOPg values in the study group of acromegaly and comparable IOPcc values in the control group [10,16]. ORA also shows IOPcc values which are less dependent on the variable biomechanical properties of the cornea compared to IOPg. Inconsistencies between IOPg and IOPcc are likely due to the effect of the stiffer cornea on IOPg measurement values. Once the influence of corneal biomechanics was eliminated, postoperative IOPcc values remained unchanged compared to preoperative values. This finding suggests that IOP values may be overestimated in acromegalic patients.

### 4.3. CCT

**Figure 2:**
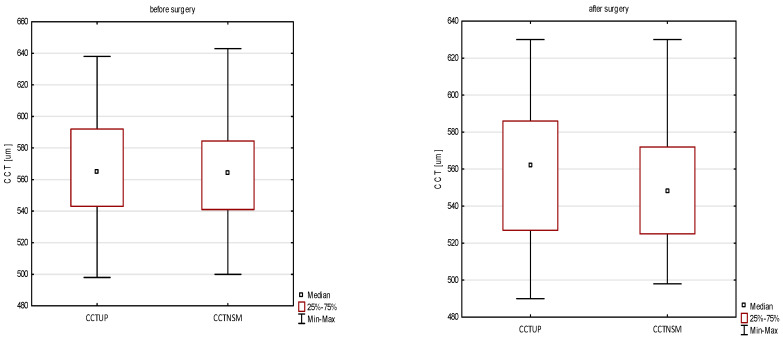
Box plot distribution of CCT_UP_ and CCT_NSM_ before and after surgery. CCT_UP_, ultrasonic pachymetry; CCT_NSM_, central corneal thickness from non-contact specular microscopy.

We observed a decrease in CCT_UP_ and CCT_NSM_ values. Before surgery, the median CCT_UP_ had been 565 μm (range from 498 μm to 638 μm) which was reduced to 562 μm (range from 490 μm to 630 μm) after surgery. Before surgery, the median CCT_NSM_ had been 563 µm (range from 500 μm to 643 μm), which then was reduced to 542 μm (range from 490 μm to 630 μm) after surgery. However, in the case of UP, the measurement values were higher as compared to CCT_NSM_, which is consistent with the tendency to indicate higher CCT values in UP (Figure 2) [29]. The UP method, considered the gold standard of corneal thickness measurement, should be the method of choice in patients with acromegaly. Compared to non-contact methods, there are no restrictions related to fixation, which may be associated with changes in the visual field caused by compression of the adenoma to the optic chiasm in patients with acromegaly [5,6,7,29]. However, further studies on corneal thickness in acromegalic patients with a rotating Scheimpflug camera or anterior segment optical coherent tomography (AS-OCT), may provide accurate and repeatable CCT readings. It has recently been shown by Kim et al. that measurements using ultrasound pachymetry, non-contact tonopchy, Pentacam HR, and Fourier-domain OCT were well correlated [30].

As regards our study, the mean value of the difference between CCT_UP_ and CCT_NSM_ was not clinically significant and amounted to 0.53 before surgery, which means that the methods provided similar results. However, the range was significant (shown in the Mountain plot chart), which may be due to the fact that the study group was not homogeneous before surgery, i.e., they differed in terms of the biochemical status (GH and IGF-1 values) and the duration of the disease. After surgery, the mean value of the difference was greater and amounted to 3.53 with the range being smaller. It may be concluded that the method of CCT measurement is less burdensome for the patient or should be used interchangeably (Figure 1).

It should be emphasized that patients with symptomatic acromegaly and significant morphological changes in the face have corneas that are thicker than in asymptomatic patients whose disease was detected accidentally on MRI, as Bramsen et al. pointed out in their study [31]. The status of disease activity—active or inactive (in biochemical remission)—is, in our opinion, of great importance as regards changes in CCT. Ciresi et al. confirmed that CCT values were higher in patients with poorly controlled acromegaly compared to the group of patients in biochemical remission, but they reported no differences in corrected IOP [12]. The researchers emphasized that higher GH, controlled/uncontrolled disease status and the duration of active disease were the risk factors for increased CCT, and the cornea itself was an organ involved in active acromegaly [12]. The correlation between CCT and the duration of acromegaly was also confirmed in another study [14].

Polat et al. reached different conclusions. They observed no difference in CCT in the study group and subgroups (active/inactive), but the group of patients included only patients treated for several years, and there were no patients with newly diagnosed diseases [15].

Studies showed that the level of IGF-1 was elevated in patients with acromegaly not only in the serum but also in the subretinal fluid and the aqueous humor [32]. Kan et al. demonstrated IGF-1 reduction in the tears of two patients with acromegaly before and after pituitary adenoma resection [33]. However, another study conducted by the same researchers revealed no differences in IGF-1 in the tears when comparing a group of patients with the control group [20]. In Sheehan’s syndrome, characterized by GH deficiency, a significant decrease in CCT was found, which positively correlated with IGF-1 levels [34]. A significant increase in CCT and IOP was demonstrated in children with GH deficiency after 12 months of GH replacement therapy [35]. The above studies suggested that the eye might be an organ affected by acromegaly and sensitive to IGF-1 and GH levels, especially in the active phase of the disease (prior to treatment for acromegaly). Our study showed a relationship between disease activity and corneal and IOP measurements.

### 4.4. IOP Measurements

**Figure 3:**
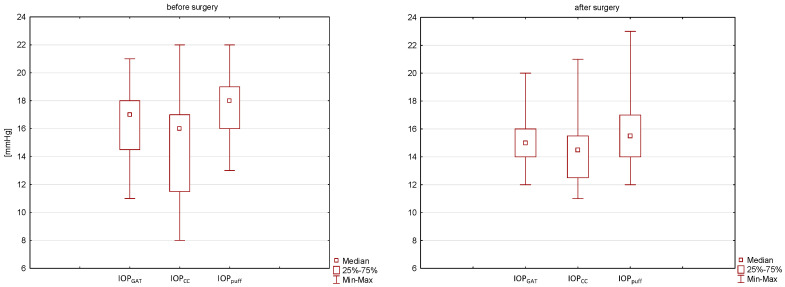
Box—plot distribution of IOP GAT, IOPc and IOP air puff before and after surgery. IOP GAT, Goldmann applanation tonometry; IOPc, intraocular pressure corrected for ultrasonic pachymetry; IOP air puff, pneumatic intraocular pressure.

A downward trend was observed after treatment in the case of IOP GAT and IOPc. Before surgery, the median IOP GAT and IOPc had been 16.2 ± 2.4 and 14.9 ± 3.9 mmHg (range from 11 and 8 mmHg to 21 and 22 mmHg) which were reduced to 15.3 ± 2.3 and 14.5 ± 2.6 mmHg (range from 12 and 11 mmHg to 20 and 21 mmHg) after surgery. However, statistical significance was not achieved, which was related to the limitations of the study, i.e., a small group size. IOPc was lower than IOP GAT both before and after surgery. Only in one patient, the intraocular pressure exceeded the statistical norm > 21 mmHg, which may predispose to glaucoma. In our study, IOP decreased statistically for the measured IOP air puff values (Figure 3). Our study is the first publication to evaluate IOP air puff in patients with acromegaly. We noted that the non-contact tonometer device tended to slightly overestimate the IOP air puff results compared to IOP GAT, similar to the results presented by Italian researchers [36]. Another publication by the same authors confirmed that CRF was the main factor influencing the difference in values between the two methods [37]. A significant reduction in CRF in patients with acromegaly may explain the clinically relevant IOP air puff result.

The results of studies assessing IOP in patients with acromegaly available in the literature vary and require more detailed analyses [9,10,11,14,15,19,20,21]. It may be due to the fact that the available studies included patients with different disease duration and not all studies categorized patients according to the biochemical status of the disease. Quaranta et al. reported significantly higher values of IOP GAT and CCT, as well as a statistically significant correlation between them [9]. However, when IOP GAT was corrected for CCT, no significant difference was confirmed with reference to the control group. It may mean that higher CCT values might lead to an overestimation of IOP GAT, which is considered the gold standard of IOP measurement [9]. Moreover, CCT is a predictive factor for identifying which patients with ocular hypertension are more likely to convert to glaucoma. Therefore, particular attention should be paid to the possibility of overestimating IOP and the possibility of changing CCT depending on disease activity in acromegalic patients [38]. An additional risk factor for glaucoma is associated with the presence of a narrower angle of the anterior chamber in acromegalic patients [11]. In turn, Sen et al. conducted a study in a larger group of patients and demonstrated that the levels of CCT, IOP GAT and IOPc were higher in acromegalic patients than in healthy subjects, and the duration of acromegaly was correlated with CCT and IOP GAT, even when the disease was hormonally controlled (GH < 1 µg/L, and no GH inhibition after oral glucose tolerance test) [13]. In contrast, Kan et al. found no differences in IOP and CCT between the acromegaly group divided into two subgroups (active/inactive) and the control group and did not report any correlation with IGF-1 in tears [19].

## 5. Study Limitations

- The group after surgery could be further divided into two parts—complete biochemical remission (IGF-1/GH normal) and maintenance treatment with somatostatin analogs, but the study groups were too small to make such a comparative analysis. We will certainly conduct further observation on a larger group of patients to assess the long-term impact of acromegaly on the eye.
- The date of the follow-up examination was not the same for all patients. The patients came from all over Poland and the long distances made it difficult to come to the control visit at the appointed time. The COVID-19 pandemic, which began in the spring of 2020, has further aggravated the problem of timely visits.
- No studies were performed to assess the tear film, corneal topography and tomography, including the analysis of the corneal epithelium, which could provide more information about the abnormalities of the eye surface and changes in the corneal structure in patients with acromegaly undergoing surgical treatment.

## 6. Conclusions

Our study revealed significant changes in biomechanics, corneal endothelium, CCT and IOP after somatotroph adenoma resection. It confirmed that the eye is sensitive to long-term overexposure to GH and IGF-1. Further research should help to clarify the extent to which changes in corneal parameters are reversible in patients in complete remission of acromegaly and in those chronically treated with somatostatin analogs.

## Figures and Tables

**Table 1:** Characteristics of the patients.

	Before Surgery			After Surgery			
	Mean ± SD	Median	Range	Mean ± SD	Median	Range	*p* Value
Age [years]	49.7 ± 13.4	49	29–75				
Gender—male [%]	35%						
Remission [%]	0%			75%			
Disease duration [years]	8.3 ± 5.2	7	2–20				
Tumor height [mm]	14.0 ± 7.4	12.5	2–33	2.8 ± 5.1	0	0–16	0.001 *
BMI	28.0 ± 4.1	27.3	21.4–38.5	27.2 ± 4.7	26.6	19.1–36.1	0.112
IGF-1 [ng/mL]	474 ± 299	359.5	102–1268	267 ± 265	173	88–1219	0.001 *
GH [µg/l]	18.2 ± 23.3	6.1	0.2–74.0	3.6 ± 6.7	1.4	0.1–30	0.001 *
Glucose [mmol/mL]	6.0 ± 0.9	5.8	5.1–9.4	5.1 ± 0.6	5	4.3–6.1	<0.001 *
BCVA	1.0 ± 0.1	1	0.8–1.0	1.0 ± 0.1	1	0.8–1.0	0.789
SE [D]	−0.46 ± 1.33	−0.38	−3.50–2.00	−0.45 ± 1.85	−0.63	−3.63–2.75	0.802
IOP air puff [mmHg]	17.7 ± 2.3	18	13–22	16.2 ± 2.9	16	12–23	0.012 *
IOP GAT [mmHg]	16.2 ± 2.4	17	11–21	15.3 ± 2.3	15	12–20	0.083
IOPc [mmHg]	14.9 ± 3.9	16	8–22	14.5 ± 2.6	14.5	11–21	0.356
CCT_UP_ [μm]	565 ± 32	565	498–638	557 ± 35	562	490–630	0.007 *

*p* value—Wilcoxon paired test, * statistically significant differences before and after surgery. SD, standard deviation; BMI, body mass index; IGF-1, insulin-like growth factor-1; GH, growth hormone; BCVA, best corrected visual acuity; SE, spherical equivalent; IOP air puff, pneumatic tonometry; IOP GAT, Goldmann applanation tonometry; CCT_UP_, central corneal thickness measured by ultrasound pachymetry; IOPc, corrected intraocular pressure. D, diopter.

**Table 2:** Corneal endothelium measurements from non-contact specular microscopy.

	Before Surgery			After Surgery			
	Mean ± SD	Median	Range	Mean ± SD	Median	Range	*p* Value
CD [cell/mm^2^]	2404 ± 214	2346	2110–2883	2525 ± 218	2532	2123–2914	0.001 *
AVG [μm^2^]	415 ± 34	416	347–473	399 ± 34	395	343–470	0.009 *
CCT_NSM_ [μm]	559 ± 34	563	500–643	547 ± 35	542	490–630	0.004 *

*p* value—Wilcoxon paired test, * statistically significant differences before and after surgery. SD, standard deviation; CD, number of endothelial cells per mm^2^; AVG, average cell size; CCT_NSM_, central corneal thickness.

**Table 3:** Ocular response analyzer measurements.

	Before Surgery			After Surgery			
	Mean ± SD	Median	Range	Mean ± SD	Median	Range	*p* Value
IOPcc [mmHg]	15.7 ± 2.7	14.9	11.4–22.8	15.1 ± 2.6	15.4	10.9–19.3	0.314
IOPg [mmHg]	15.8 ± 3.4	14.9	11.9–25.8	14.4 ± 2.8	14.2	8.8–20.2	0.011 *
CH [mmHg]	10.9 ± 1.4	10.8	8.4–14.3	10.3 ± 1.4	10.0	8.8–13.6	0.016 *
CRF [mmHg]	11.0 ± 1.8	10.3	8.9–15.3	10.0 ± 1.6	9.7	7.9–14.4	0.001 *

*p* value—Wilcoxon paired test, * statistically significant differences before and after surgery. IOPcc, corneal-compensated intraocular pressure; IOPg, Goldmann-correlated intraocular pressure; CH, corneal hysteresis; CRF, corneal resistance factor.

**Table 4:** Correlation analyses between GH, IGF-1, glucose, tumor height and ORA parameters in the study group before and after treatment.

	GH				IGF-1				Glucose				Tumor Height	
ORA	Before Surgery		After Surgery		Before Surgery		After Surgery		Before Surgery		After Surgery		Before Surgery	
	r	*p*	r	*p*	r	*p*	r	*p*	r	*p*	r	*p*	r	*p*
CH	0.373	0.116	0.038	0.878	0.508	0.026	−0.020	0.936	0.634	0.004	0.153	0.532	0.624	0.004
CRF	0.394	0.095	0.279	0.248	0.548	0.015	−0.023	0.926	0.548	0.015	0.346	0.146	0.573	0.010
IOPg	0.317	0.186	0.399	0.091	0.365	0.124	0.080	0.745	0.282	0.242	0.515	0.024	0.218	0.371
IOPcc	0.239	0.325	0.239	0.325	0.208	0.394	−0.018	0.943	−0.078	0.752	0.355	0.136	0.225	0.355

Spearman correlation formula; GH, growth hormone; IGF-1, insulin-like growth factor-1; ORA, ocular response analyzer; IOPcc, corneal-compensated intraocular pressure; IOPg, Goldmann-correlated intraocular pressure; CH, corneal hysteresis; CRF, corneal resistance factor.

## Data Availability

The data that support the findings of this study are available upon request from the corresponding author.
